# Protocol for culturing the endocrine junctional zone of the mouse placenta in serum-free medium

**DOI:** 10.1016/j.xpro.2023.102384

**Published:** 2023-06-27

**Authors:** Hong Wa Yung, Graham J. Burton, D. Stephen Charnock-Jones

**Affiliations:** 1 Department of Obstetrics and Gynaecology, University of Cambridge, Level 2, The Rosie Hospital Cambridge Biomedical Campus, Robinson Way, Cambridge CB2 0SW, UK; 2 Centre for Trophoblast Research, University of Cambridge, Physiological Laboratory, Downing Street, Cambridge CB2 3EG, UK

**Keywords:** Cell culture, Developmental biology, Model Organisms, Proteomics

## Abstract

The lack of a suitable explant culture system restricts the study of factors secreted by the mouse placenta into maternal circulation. Here, we present a protocol for culturing the endocrine junctional zone of the mouse placenta, free from the decidua and labyrinthine layers in serum-free medium. We describe steps for dissecting and separating layers, dicing tissue, and culture setup. We then detail medium processing for downstream analysis. This model allows the investigation of placental signals that may regulate maternal physiology.

For complete details on the use and execution of this protocol, please refer to Yung et al. (2023).^1^

## Highlights

• We have developed a method for the culture of the junctional zone of mouse placenta
• Serum-free culture allows the study of secreted proteins
• Culture under physiological oxygen conditions (10%) reduces cell stress

## Abstract (editor highlights)

Publisher’s note: Undertaking any experimental protocol requires adherence to local institutional guidelines for laboratory safety and ethics.

## Graphical abstract

**Figure:**
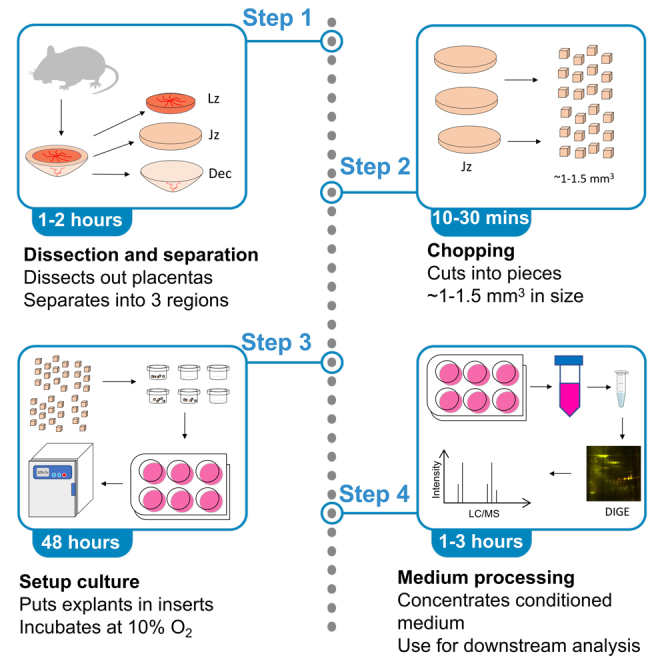

## Before you begin

**Timing: 13–16 days (for step 1)**

**Timing: 0.5–3 h (for step2)**

1. Set up a timed mating: generate a pregnant female for collection of placentas at embryonic day 12.5 (E12.5).
  a. Put male and virgin female mice (∼10–16 weeks old) together, check for vaginal plug the following and subsequent mornings.
  b. A pregnant female is identified on the basis of presence of a vaginal plug. Noon of the day that the vaginal plug is identified, is defined as E0.5.
  c. Separate the pregnant female from the male and house for an additional 12 days to reach a gestational age of E12.5.
2. Preparation of reagents and defined Junctional zone (Jz) explant medium (Jz-medium).
  a. Prepare and autoclave phosphate buffered saline (PBS).
  b. Prepare defined Jz-medium for culture.
  c. Prepare 70% ethanol for disinfection spray.
  d. Autoclave dissecting instruments and glassware. Alternatively, uses sterile plasticware.

### Institutional permissions

All animal work was performed under the authority granted by the Animals (Scientific Procedures) Act 1986 Amendment Regulations 2012 and following ethical review by the University of Cambridge Animal Welfare and Ethical Review Body.

***Note:*** Use of animals must be performed in accordance with relevant institutional and national guidelines and regulations.

## Key resources table

**Table:** 

REAGENT or RESOURCE	SOURCE	IDENTIFIER
**Chemicals, peptides, and recombinant proteins**		

Oxoid™ Phosphate Buffered Saline Tablets	Thermo Scientific	BR0014G
Ethanol absolute	Merck Life Science	1070172511
3-(4,5-Dimethylthiazol-2-yl)-2,5-diphenyl tetrazolium bromide (MTT)	Thermo Scientific	M6494
Advanced DMEM/F12 medium	Invitrogen	12634010
GlutaMax (100×)	Invitrogen	35050061
B27 supplement (50×)	Invitrogen	17504044
Penicillin/Streptomycin (100×)	Invitrogen	15140-122

**Experimental models: Organisms/strains**		

C57BL/6 mice, wild-type, pregnant at embryonic day 12.5 (E12.5)		N/A

**Other**		

O_2_ / CO_2_ incubator	Sanyo	MCO-5M
Stereo microscope (>10× magnification)	Lecia	Wild-M10
Blunt forceps	Fisher scientific	15576579
Ultrafine forceps	Fine Science Tools	11370-40
Fine forceps	Fine Science Tools	11413-11
Blunt scissors	Fisher scientific	10208930
Fine scissors	Fisher scientific	12952055
35 mm dish	Corning	353001
100 mm dish	Corning	353003
Gosselin™ Straight Container with Red Screw Cap	Fisher scientific	12377547
Netwell™ (diameter 24 mm, mesh size 74 μm (for 6 well plate)	VWR	734-1588
Netwell™ (diameter 15 mm, mesh size 74 μm (for 12 well plate)	VWR	734-1586

## Materials and equipment

**Table:** Defined Jz-medium

Reagent	Final concentration	Amount
Advanced DMEM/F12 medium	N/A	485 mL
GlutaMax (100×)	1×	5 mL
B27 supplement (50×)	0.5×	5 mL
Penicillin/Streptomycin (100×)	1×	5 mL
Total	N/A	500 mL

Store at 4°C for a maximum period of 1 month.

• 1× PBS solution (autoclaved): add 5 tablets in 500 mL Milli-Q H_2_O and autoclave.

Store at 4°C for a maximum period of 1 year.

• 70% ethanol: mix 700 mL of ethanol absolute, with 300 mL ddH_2_O.

Store at room temperature for a maximum period of 1 year.

• 50 mg/mL MTT solution: dissolve 50 mg MTT (3-(4,5-dimethylthiazol-2-yl)-2,5-diphenyl tetrazolium bromide) in 1 mL sterile Milli-Q H_2_O and vortex.

Store at 4°C for a maximum period of 6 months.

Dissection of the uterine horns from the animal can be carried on a clean bench in a clean dissecting room. Ideally the separation of the placenta from amniotic sac and dissection of the junctional zone and dicing the junctional zone should be carried out in a Class I microbiological safety cabinet (MSC). It is possible to use a Class 2 MSC but adaptations are needed to house the dissecting microscope. Alternatively, these steps can be carried out on a disinfected (70% ethanol) bench in tissue/cell culture room with minimal air movement (i.e., using a bench away from doors and windows with any air conditioning switched off and avoiding opening and closing the door).

A humidified 37°C incubator capable of maintaining an oxygen concentration of 10%, and carbon dioxide concentration of 5% is necessary.

**CRITICAL:** As the Jz will be cultured, steps must be taken to avoid bacterial and fungal contamination. Good laboratory practice and antiseptic technique are essential to minimize contamination. Use of the anti-mitotic agent and/or additional antibiotics are the last resort for suppressing microbial growth as some of these agents may induce low-grade cellular stress.

Culture under ambient atmospheric oxygen causes cell stress, hence the more physiological level of 10% is used.

***Optional:*** Conditioned medium is harvested and can be concentrated using a Vivaspin (5000 MWCO) centrifugal concentrator (2 mL, 6 mL or 20 mL, Vivaproducts Inc., USA) depending on the volume of the medium. A refrigerated centrifuge for 15 mL and/or 50 mL tubes (Cat# 5810R, Eppendorf, UK) is required.

## Step-by-step method details

**Timing: approximately 3 h**

The section describes the step-by-step procedures to setup the Jz explants culture. This includes taking out the uterine horns from pregnant mouse, dissecting out placenta, separating placenta into different regions and setting up the explant culture.

### Preparation of junctional zone explants culture – Day 1

**Timing: 1–2 h (for step 1)**

**Timing: ∼10–30 min (for step 2)**

**Timing: ∼10 min (for step 3)**

**Timing: 48 h (for step 4)**

**Figure 1:**
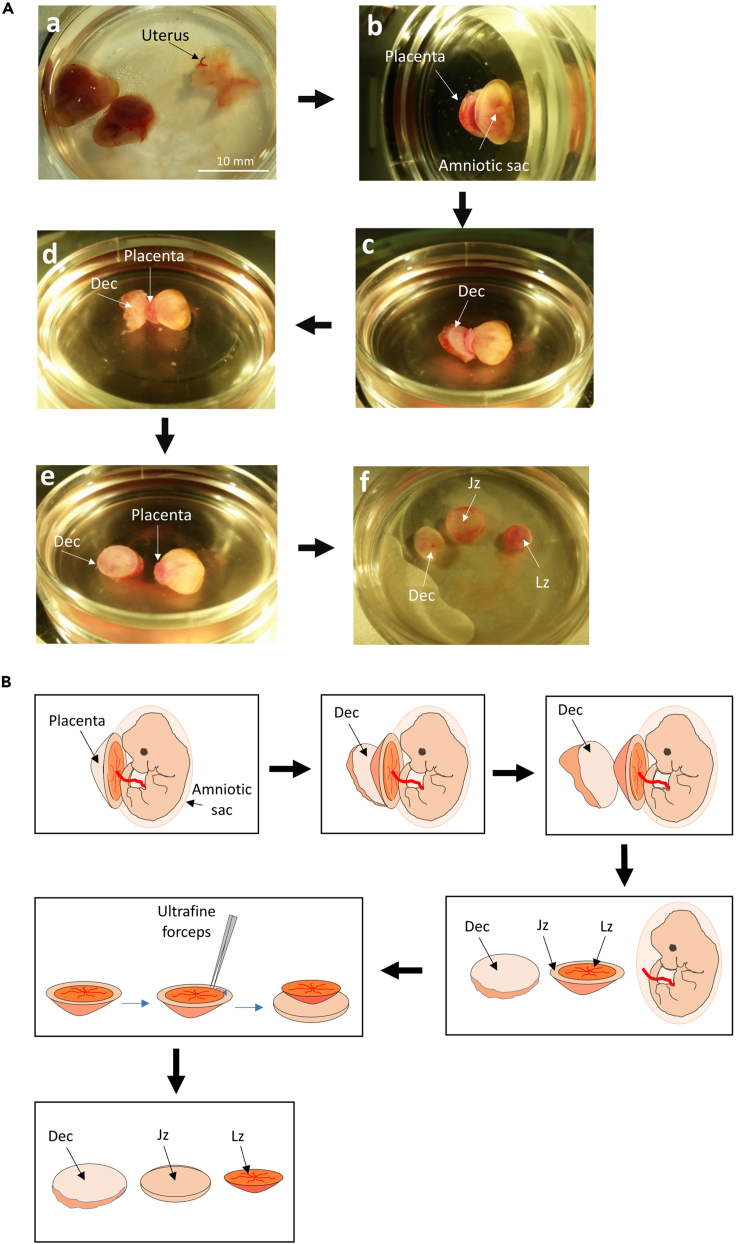
Step by step illustrations of how to dissect off the amniotic sac before "peeling" away maternal decidua layer and separating into the junctional and labyrinth zones (A) (a) Dissecting the amniotic sac out of uterus. (b–e) Step by step shows "peeling" off decidual layer from the placenta. The procedure starts at the edge and gradually moves towards the center of the disc. (f) The dissected decidua, junctional zone and labyrinth. Scale bar =10 mm (B) Schematic diagram illustrating the steps in (A).

***Note:*** If the placentas are from animals with the same genotype the same dish containing Jz-medium may be re-used. Otherwise clean forceps and new inserts are required for each placenta.

***Note:*** Clean the forceps by dipping in 70% ethanol following by serial rinsing in 3 pots PBS to remove residual ethanol.

1. Dissecting out junctional zone from placenta
  ***Note:*** All dissecting instruments used below must be sterile or autoclaved
  a. Removal of uterine horns from the pregnant animal at E12.5.
    We have not tested the procedure at other gestational ages. However, the junction zone decreases in size after E16.5.^2^
    i. Cull the pregnant animal by cervical dislocation.
    ii. Sterilize the abdomen of the animal with 70% ethanol, blot dry with clean paper towel to remove excess ethanol.
    iii. Make a skin incision in the abdomen with blunt scissors and the skin peeled back.
      ***Note:*** This can be done by hand or using blunt scissors.
      ***Note:*** Take care to prevent the outer skin surface touching the abdominal muscle and so avoid possible cross-contamination.
    iv. Use a new pair of blunt scissors and a pair of blunt forceps to open up the abdomen.
    v. Remove the whole uterus and quickly submerge in 50 mL (per animal) ice-cold PBS in a Gosselin container and close the cap.
      ***Note:*** Keep the container on ice during transport.
      ***Note:*** Take the uteri to the Class I MSC (or the bench as described above) for the following steps.
  b. Carefully separate Individual implantations containing the embryo and placenta from the uterine horns and keep under ice-cold PBS in a sterile dish (Figure 1A).
    ***Note:*** Use sufficient PBS to submerge the entire implantation.
    i. Prepare a 35 mm dish containing ice-cold PBS for each implantation and leave it on the top of the ice.
    ii. Using blunt forceps take the uterine horns out of the container and place in a 100 mm dish containing ice-cold PBS and rinse briefly to remove material loosely attached to the tissue.
    iii. Using blunt forceps, grasp the junction between the two uterine horns and remove from the PBS.
    iv. Using fine scissors separate the implantations from the uterine horns by cutting between each one.
      ***Note:*** The myometrium may contract and expel the amniotic sac.
    v. Place each implantation in a separate 35 mm dish with cold PBS prepared in step b.i. above. Place the lid on to minimize contamination.
    vi. Keep the dish with the implantation, amniotic sac and placenta on ice until the next step.
  c. Separating the maternal decidua and dissecting out the labyrinth region are performed under a stereo-microscope (Figure 1A(b–e). For a better view, a schematic diagram of the images in Figure 1A is presented in Figure 1B.
    i. Process one implantation at a time in the 35 mm dish in ice-cold PBS.
    ii. Using fine forceps, first carefully remove the remaining uterine tissue still enclosing amniotic sac and placenta. If necessary, uses forceps to gently tear the uterine tissue apart.
      ***Note:*** Occasionally, the decidua separates from the placenta and adheres to the myometrium. If this happens, jump to step d below.
    iii. Using the same fine forceps, peel off the decidua layer from the basal (outer convex) surface of the placenta. Use the amniotic sac as an anchor to hold the placenta in position.
    iv. Access the decidual layer from the edge of the placenta.
    v. Peel off from the edge of the placenta gently and gradually extend towards the center of the disc to remove entire decidua layer (Figures 1C and 1D).
    vi. Once the decidual layer is removed, separate the amniotic sac from the placenta.
      ***Note:*** At the position where the umbilical cord connects to the placenta and the embryo, use the fine forceps to cut the umbilical cord as close as possible to the chorionic plate of the placenta in order to minimise chorion/amnion contamination.
  d. Carefully dissect the labyrinth from the junctional zone with ultrafine forceps.
    i. Lay the placenta flat with chorionic surface with umbilical cord attached facing upwards.
      ***Note:*** Identify the labyrinth as the central red area as it contains maternal and fetal blood vessels. The junctional zone is the paler, almost colourless rim.
    ii. Use one forceps to gently hold the placenta and use another forceps to separate along the edge of the labyrinth. Push closed forceps into the boundary between the two and allow them to open slowly to tease the tissue apart. Continue around the disc.
    iii. After completing the round, flip the placenta over. Use one set of forceps to lift up the edge of junctional zone layer and the other forceps to slowly tease apart the two layers towards the center of the disc until the two regions are completely separated.
      ***Note:*** If necessary, clean up the dissected junctional zone by removing any remaining labyrinth tissue which is pink in colour (Figure 1F).
    iv. Transfer the junctional zone to a new dish containing pre-cooled DMEM/F12 medium and keep on top of the ice until step 2).
2. Dicing junctional zone tissue.
  a. Dice the dissected junctional zone tissue into cubes approximately 1–1.5 mm^3^ in size using fine forceps inside an insert in dish containing the 2 mL Jz-medium under the stereo-microscope.
  b. Place the junctional zone tissue from one or two placenta in a 15 mm or 24 mm diameter Netwell™ respectively.

3. Set up *ex vivo* junctional zone explant cultures
  a. Transfer the 15 mm or 24 mm diameter inserts containing junctional zone explants into either a 12-well plate or a 6 well plate with well containing 1 mL or 3 mL Jz-medium respectively.

***Note:*** Make sure all tissue explants are submerged under the medium.

**Figure 2:**
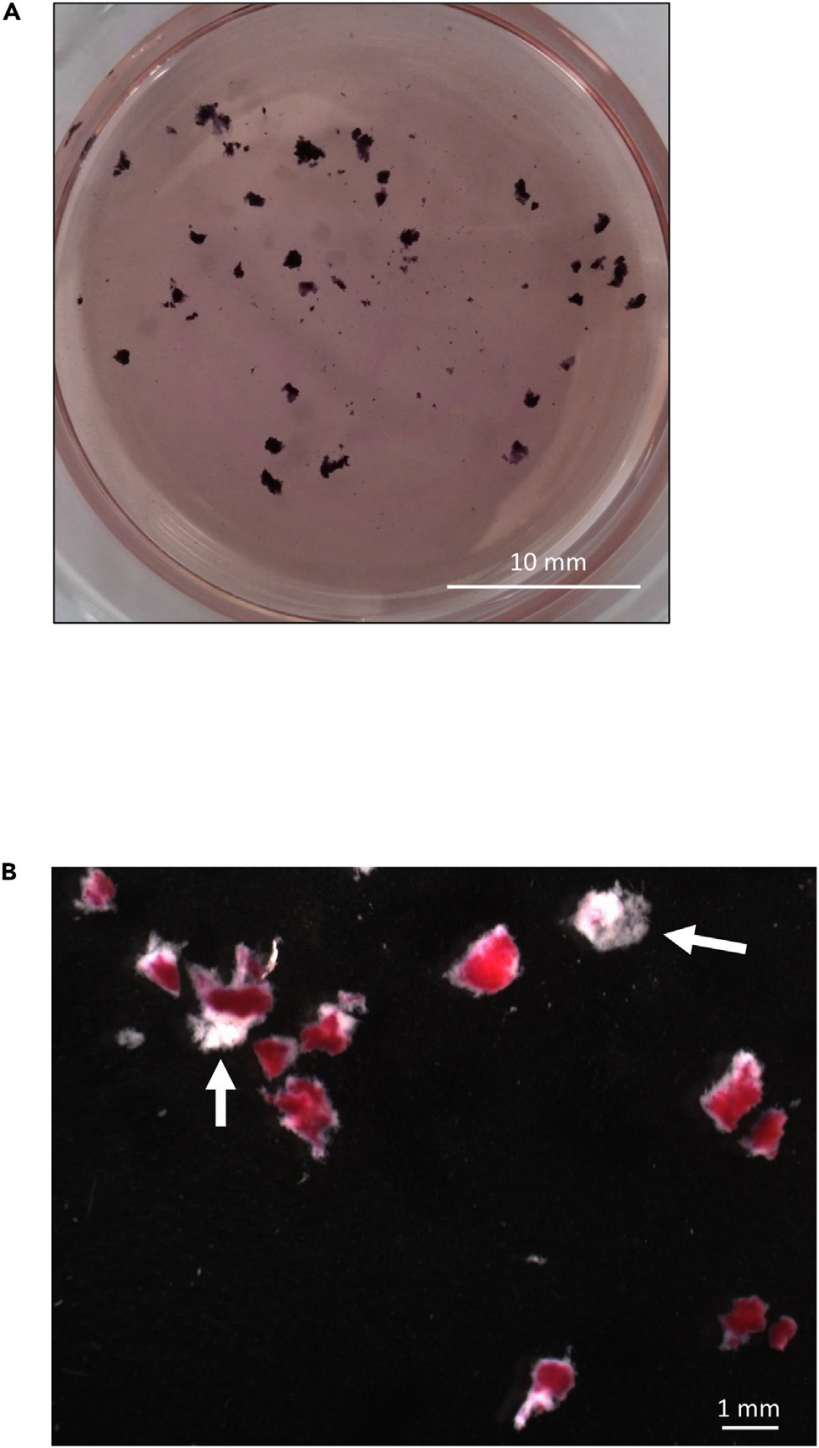
The junctional zone explants are viable after 48 h culturing After 48 h culturing, MTT was added to the culture medium at a final concentration of 500 μg/mL and incubated in the incubator for 1 h. The dark blue staining indicates viability of the tissues. (A) Low magnification. Scale bar = 10 mm. (B) High magnification. Arrows show the explants that do not show positive MTT staining, indicating the tissue is dead or at the very least, has lost mitochondrial function. Scale bar = 1 mm.

4. Culture incubation
  a. Incubate the plate in a humidified 37°C incubator containing 5% CO_2_ and 10% O_2_ balance N_2_ for 48 h.
  b. Check viability of the tissue by adding MTT (50 mg/mL) to the culture medium at a final concentration of 500 μg/mL. After incubation for 1 h in the incubator dark blue/purple staining indicates viable tissues (Figure 2). In the higher magnification image, a few explants are not stained after MTT incubation (Figure 2B, arrows).

### Collecting conditioned medium for downstream analysis – Day 3

**Timing: Approximately 3 h**

**Timing: 1–3 h (for step5)**

This section describes step by step procedures for collecting and processing conditioned medium and harvesting explant tissues ready for downstream analysis.

5. Collecting and concentrating conditioned medium
  a. At the end of incubation, collect the junctional zone explant tissue from the inserts and place in a 1.5 mL tube and snap freeze in liquid nitrogen for subsequent analysis (Western blotting, PCR etc).
  b. For the conditioned medium (CM) from each well, it can either collect separately or pool together in 15 mL or 50 mL centrifuge tubes. Centrifuge the CM at 4000 rpm (1771 g) for 30 min at 4°C to remove tissue debris.
  c. Transfer the supernatant (free of cell debris) to a new tube.
  d. If the clarified CM is to be concentrated, transfer it to a Vivaspin centrifugal concentrator and centrifuge according to the manufacturer’s instructions until the desired reduction in volume is achieved. Transfer the concentrated CM to a 1.5 mL tube.
    ***Note:*** The CM is generally concentrated by 20 to 30-fold. Optimization is required to identify correct extent of concentration for different downstream processes.
  e. Store both junctional zone explant tissue fragments and concentrated conditioned medium at -80°C until further analysis.
    **CRITICAL:** To avoid any contamination during explant culture, it is crucial to keep the entire procedure as clean as possible. All plasticware, glassware and instruments must be sterilized prior to use. Equipment and bench surfaces need to be disinfected with 70% ethanol prior to use.
    **CRITICAL:** Keep lids/caps of all dishes and tubes closed whenever possible to minimise contamination. This is especially important when performing these procedures on the open bench.
    **CRITICAL:** Keep placentas and dissected junctional zone under ice-cold PBS throughout the dissection procedure to minimise cellular stress.

## Expected outcomes

A successful junctional zone explant culture should show low contamination of labyrinth tissue. This can be judged by real-time quantitative reverse transcription polymerase chain reaction analysis of Jz and Lz specific markers such as *Tpbpa* and *Gcm1* respectively. There should be an increase of survival cell signaling such as increased phosphorylation of Akt, a survival, growth and proliferation kinase, while reduced cellular stress markers such as phosphorylation of p38 kinase, Jnks and heat shock proteins, in cultured tissues at 48 h compared to the tissues collected at time 0 (before culture). Tissue fragments should be stained dark blue after incubation with MTT. The cultures should also show no sign of bacterial and fungal contamination. These results were presented in supplementary figure S5 in Yung et al., 2023.^1^

## Limitations

We also have not tested extending the incubation beyond 48 h so do not know whether it is suitable for prolonged culture. We did not test the effect of changing the culture medium during culture.

This junctional zone explant model can only be used for placenta containing distinct junctional and labyrinth zones. Therefore, it is likely to be suitable for studying the rat placenta, but it will not be suitable for other species.

We have not tested this method using placentas at gestations later than E12.5. It is known that the labyrinth volume increases as gestation advances, while junctional zone and decidua increase their volume and peak at E16.5 before declining at E18.5.^2^ Nonetheless, we anticipate it may be useful for studies at other gestational ages.

## Troubleshooting

### Problem

Contamination of cultures.

### Potential solution

Good general laboratory practice is essential. Ensure the room used for dissecting the animal is clean and does not have excessive air movement (such as from open windows or directly under ventilation openings). Dissection of the junctional zone with the stereo-microscope can be carried out in a Class 1 MSC. Dicing the junctional zone into small pieces can be similarly carried out inside a Class 1 MSC. If contamination persists, avoid movement inside the room while performing the dissection. Alternatively, the dissection can be carried out in a Class 2 MSC. Use of additional antibiotics including anti-mitotic agents is the last resort.

### Problem

Low tissue viability or high cellular stress in explants.

### Potential solution

This may result from prolonged dissection time and/or the PBS used not being ice cold. PBS should be pre-cooled prior to use. It should then be kept in an ice-bath at all times. Spongiotrophoblast cells in the junctional zone are highly metabolically active. If the placenta is kept in PBS for a prolonged period because of slow procedures, this may cause nutrient deprivation, thereby provoking stress. The PBS being not sufficiently cold may also facilitate higher metabolic activity in tissues, contributing to stress. The cellular stress levels in the explants can be checked using western blotting analysis for cellular stress markers such as heat shock proteins, ER and mitochondrial chaperone levels on Time = 0 tissues. There are multiple ways to shorten the procedure time, such as dissecting fewer placentas. For a beginner, it may take 5–10 min to dissect out the junctional zone from a single placenta. However, once you become familiar with the technique, it usually takes 2–3 min. Ideally, limit the entire procedure time to under 2 h.

## Resource availability

### Lead contact

Further information and requests for resources and reagents should be directed to, and will be fulfilled by the lead contact, Prof. Steve Charnock-Jones (dscj1@cam.ac.uk).

### Materials availability

All reagents and consumables are commercially available.

### Data and code availability

Not applicable.
